# Assessing the added value of apparent diffusion coefficient, cerebral blood volume, and radiomic magnetic resonance features for differentiation of pseudoprogression versus true tumor progression in patients with glioblastoma

**DOI:** 10.1093/noajnl/vdad016

**Published:** 2023-02-21

**Authors:** Riccardo Leone, Hagen Meredig, Martha Foltyn-Dumitru, Felix Sahm, Stefan Hamelmann, Felix Kurz, Tobias Kessler, David Bonekamp, Heinz-Peter Schlemmer, Mikkel Bo Hansen, Wolfgang Wick, Martin Bendszus, Philipp Vollmuth, Gianluca Brugnara

**Affiliations:** Department of Neurology, University of Bonn, Bonn, Germany; Department of Neuroradiology, Heidelberg University Hospital, Heidelberg, Germany; Division for Computational Neuroimaging, Heidelberg University Hospital, Heidelberg, Germany; Department of Neuroradiology, Heidelberg University Hospital, Heidelberg, Germany; Division for Computational Neuroimaging, Heidelberg University Hospital, Heidelberg, Germany; Department of Neuroradiology, Heidelberg University Hospital, Heidelberg, Germany; Division for Computational Neuroimaging, Heidelberg University Hospital, Heidelberg, Germany; Department of Pathology, Heidelberg University Hospital, Heidelberg, Germany; Department of Pathology, Heidelberg University Hospital, Heidelberg, Germany; Department of Radiology, German Cancer Research Center (DKFZ), Heidelberg, Germany; Department of Neurology, Heidelberg University Hospital, Heidelberg, Germany; Department of Radiology, German Cancer Research Center (DKFZ), Heidelberg, Germany; Department of Radiology, German Cancer Research Center (DKFZ), Heidelberg, Germany; Center for Functionally Integrative Neuroscience (CFIN), University of Aarhus, Aarhus, Denmark; Department of Neurology, Heidelberg University Hospital, Heidelberg, Germany; Department of Neuroradiology, Heidelberg University Hospital, Heidelberg, Germany; Department of Neuroradiology, Heidelberg University Hospital, Heidelberg, Germany; Division for Computational Neuroimaging, Heidelberg University Hospital, Heidelberg, Germany; Department of Neuroradiology, Heidelberg University Hospital, Heidelberg, Germany; Division for Computational Neuroimaging, Heidelberg University Hospital, Heidelberg, Germany

**Keywords:** glioblastoma, machine-learning, perfusion-MRI, pseudoprogression, radiomics

## Abstract

**Background:**

Pseudoprogression (PsPD) is a major diagnostic challenge in the follow-up of patients with glioblastoma (GB) after chemoradiotherapy (CRT). Conventional imaging signs and parameters derived from diffusion and perfusion-MRI have yet to prove their reliability in clinical practice for an accurate differential diagnosis. Here, we tested these parameters and combined them with radiomic features (RFs), clinical data, and MGMT promoter methylation status using machine- and deep-learning (DL) models to distinguish PsPD from Progressive disease.

**Methods:**

In a single-center analysis, 105 patients with GB who developed a suspected imaging PsPD in the first 7 months after standard CRT were identified retrospectively. Imaging data included standard MRI anatomical sequences, apparent diffusion coefficient (ADC), and normalized relative cerebral blood volume (nrCBV) maps. Median values (ADC, nrCBV) and RFs (all sequences) were calculated from DL-based tumor segmentations. Generalized linear models with LASSO feature-selection and DL models were built integrating clinical data, MGMT methylation status, median ADC and nrCBV values and RFs.

**Results:**

A model based on clinical data and MGMT methylation status yielded an areas under the receiver operating characteristic curve (AUC) = 0.69 (95% CI 0.55–0.83) for detecting PsPD, and the addition of median ADC and nrCBV values resulted in a nonsignificant increase in performance (AUC = 0.71, 95% CI 0.57–0.85, *P* = .416). Combining clinical/MGMT information with RFs derived from ADC, nrCBV, and from all available sequences both resulted in significantly (both *P* < .005) lower model performances, with AUC = 0.52 (0.38–0.66) and AUC = 0.54 (0.40–0.68), respectively. DL imaging models resulted in AUCs ≤ 0.56.

**Conclusion:**

Currently available imaging biomarkers could not reliably differentiate PsPD from true tumor progression in patients with glioblastoma; larger collaborative efforts are needed to build more reliable models.

**Key Points**

- Different imaging techniques have been proposed for the noninvasive detection of PsPD on MRI.
- ADC, CBV and radiomic MR features could not reliably distinguish PsPD from PD in our cohort.
- Imaging features did not improve models built with clinical data and MGMT methylation status.

**Importance of the Study**

Differentiation of PD from PsPD still represents a major diagnostic dilemma in the follow-up of glioblastoma patients treated with chemoradiotherapy, as the 2 phenomena are seldom distinguishable with conventional imaging signs. Several imaging techniques have been proposed to address this issue, also combined through machine- or deep-learning algorithms, with variable results. We assessed the impact of ADC, CBV and radiomic MR features to noninvasively differentiate PsPD from PD with an automated and fully reproducible analysis using machine- and deep-learning-based models and with stringent patient selection criteria. We found that currently available imaging biomarkers were not sufficient to reliably differentiate PsPD from PD in our patient cohort and did not improve simpler models which included only clinical information and MGMT promoter methylation status. Our study suggests that novel imaging techniques or larger, multi-institutional studies are needed to potentially obtain an accurate noninvasive detection of PsPD.

Glioblastoma represents the most common primary brain tumor,^1^ and it is still associated with a dismal prognosis; despite repeated trials with novel therapeutic approaches, the gold standard of therapy still consists of maximal safe surgical resection plus radiotherapy (RT) and concomitant chemotherapy with temozolomide.^2,3^ One of the main diagnostic challenges in the posttherapeutic evaluation of these patients is the reliable discrimination of pseudoprogression (PsPD) from true progressive disease (PD) on follow-up magnetic resonance imaging (MRI) studies. According to the Response Assessment Criteria in Neuro-Oncology (RANO) criteria, the diagnosis of PD is not possible in the first 12 weeks after the end of RT, unless there is a clear new enhancement outside of the radiation field (beyond the 80% isodose line) or if there is unequivocal evidence of viable tumor on histopathologic sampling.^4^ PsPD radiologically mimics PD, but the increase in tumor enhancement or T2-FLAIR signal alteration later stabilizes or subsides without any change in therapy.^5^ Thus, at present, only the longitudinal evaluation of these patients or an invasive evaluation with tissue sampling through biopsy can discriminate between PsPD and PD. The resulting diagnostic delay may have important clinical significance: on the one hand, failure to recognize patients with PsPD may result in premature discontinuation of an effective treatment,^6^ on the other hand, including those patients in clinical trials may lead to biased results of the efficacy of a subsequent treatment and it is currently not recommended.^4,6^

Although the pathological mechanisms underlying PsPD and PD are different—with the former thought to arise from inflammatory transient blood-brain barrier disturbances and the latter from the neoplastic blood-brain barrier disruption due to tumor cells proliferation and invasion^5^—these 2 phenomena are often indiscernible with conventional imaging signs.^7^ Moreover, they may also occur simultaneously in a pathological continuum due to the diffusely infiltrating nature of GB tumor cells and the suboptimal disease control of the currently available therapies. In fact, mixed responses on histopathological samples combining different ratios of inflammation and tumor proliferation are found in up to 28% of patients.^8^ The occurrence of PsPD has been associated with the O(6)-methylguanine-DNA methyltransferase (MGMT) promoter methylation and the isocitrate dehydrogenase (IDH) mutation status, but neither of these molecular information nor other available demographic data appear self-sufficient to reliably predict a possible PsPD.^9,10^

To increase the reliability of noninvasive PsPD diagnosis, several studies have evaluated the benefits of physiologic imaging parameters, mainly the apparent diffusion coefficient (ADC) derived from diffusion-weighted imaging (DWI) and the relative cerebral blood volume (rCBV) calculated from dynamic-susceptibility contrast (DSC).^11,12^ These techniques have shown good diagnostic performances in a previous meta-analysis,^13^ with a pooled sensitivity of 71% (95% CI 60–80) and specificity of 87% (95% CI 77–93) for ADC and sensitivity of 87% (95% CI 82–91) with a specificity of 86% (95% CI 77–91) for rCBV. Nonetheless, the studies analyzed in this meta-analysis presented different ground truth references and patient inclusion criteria, possibly undermining the estimation of the actual diagnostic performance of ADC and normalized relative cerebral blood volume (nrCBV) (eg, histopathological sampling vs imaging follow-up); current evidence is also relatively low, and mainly based on small single-center retrospective studies or limited patient series. Advanced computational techniques, such as radiomics, combined with machine learning (ML) or deep learning (DL) approaches have also been tested in an attempt to probe the spatial characteristics of tissues at a smaller level compared to what is achievable by the human eye.^8,14–18^ Many of these studies however also suffer from some of the same limitations which prevent generalizability of the models, such as small sample sizes or lack of sufficient external validation.

Our objective was to comprehensively test the currently available imaging techniques in a stringently selected patient cohort and with fully reproducible methods; we aimed to develop ML and DL classifiers based on different combinations of anatomical and physiologic MRI sequences, also taking into consideration routine demographic and clinical data and MGMT promoter methylation status, with the aim to discriminate between early PD and PsPD at the first occurrence in patients with IDH-wildtype glioblastoma treated with the standard chemoradiotherapy regimen.

## Material and Methods

All investigations were conducted in accordance with the Declaration of Helsinki. Retrospective evaluation of imaging data and health electronic records were approved by the local ethics committees and informed consent was waived (S-784/2018). Sample size was based on data availability and not preestablished.

### Participants

All consecutive patients with histologically and/or methylation assay confirmed glioblastoma according to the 2016 WHO classification^19^ (*n* = 1178) who underwent imaging follow-up at the Department of Neuroradiology of the Heidelberg University Hospital (Heidelberg, Germany) and/or the Department of Radiology of the German Cancer Research Center (DKFZ, Heidelberg, Germany) and clinical follow-up and treatment at the Department of Neurology of the Heidelberg University Hospital (Heidelberg, Germany) and/or the National center for tumor diseases (NCT, Heidelberg, Germany) between January 2010 and April 2021 were screened for this study.

Inclusion criteria in the present analysis were: age > 18 years old; IDH-wildtype status; availability of information regarding MGMT promoter methylation status (methylated/nonmethylated); concurrent chemoradiotherapy according to the Stupp protocol (Temozolomide + RT with 60 Gy of total dose) ± adjuvant proton therapy; availability of a postoperative MRI performed 72 hours after surgery to use as baseline; an increase of more than 25% in the sum of the products of perpendicular diameters of a contrast enhancement (CE) lesion measured on postcontrast T1-weighted MR images (cT1) or a new CE lesion inside the high-dose RT field occurring in the first 7 months after the end of RT. The evaluated time period was set to 7 months after the end of RT, following the results of a previous study conducted at our institution, in which PsPD suspicion was recorded in 11.1% of patients until 7 months after chemoradiotherapy and no other occurrences were observed at later timepoints.^20^ The inclusion flowchart is depicted in Supplementary Figure 1, and exclusion criteria are further listed in the Supplementary Materials. Briefly, we excluded patients who underwent biopsy (*n* = 32), as well as with missing clinical information regarding treatment (*n* = 362), varying treatment regimens (also comprising targeted treatment to vascular growth factors) (*n* = 409), noncompliance with RANO imaging criteria for PsPD (*n* = 204) or insufficient quality in the imaging data (*n* = 46).

### Outcomes

Suspected imaging PsPD were classified according to the RANO criteria.^4^ Namely, patients presenting at follow-up with either (1) an increase in the lesion CE (product or sum products of perpendicular diameters) of at least 25% of a measurable lesion (> 10 × 10 mm in 2 perpendicular diameters) or (2) a new nodular component ≥10 mm, both within the radiation field and in the first 7 months after the end of RT as compared to the baseline MRI scan, were considered as suspected for early progressive disease.

Histological confirmation of PsPD is not yet regularly performed in clinical practice and was therefore not available for most patients. The classification of patients into either true PD or PsPD was thus set through the evaluation of follow-up MRI scans, as previously reported.^21^ True PD was defined as a further lesion increase at 1 of the 2 subsequent follow-up MRI scans (performed at least 4 weeks after the MRI of initial suspicion) or as a tumor-related fatal outcome within 6 months after the initial enhancement increase. Patients were classified as PsPD if the initial enhancement increase was stable or reduced within the 2 subsequent follow-up MRI scans and in absence of death at 6 months.

### Predictors: Demographic, Clinical Data, and MGMT Promoter Methylation Status

For each patient, the following demographic and clinical variables—collectively referred to as clinical data for simplicity of reading—were recorded and used to build the predictive models: age, sex, use of proton therapy in addition to traditional chemoradiotherapy (y/n), extent of surgical resection (subtotal/gross-total resection), and time from the end of RT to the first appearance of an MRI questionable lesion. MGMT promoter methylation status (categorical variable: methylated/nonmethylated) was also recorded.

### Image Acquisition

Images were acquired in the routine clinical workup using a 3 Tesla MR system (Magnetom Verio/Trio TIM/Prisma Fit/Skyra, Siemens Healthcare) with a 12-channel head-matrix coil. Briefly, the protocol included T1-weighted 3D MPRAGE images both before (T1) and after (cT1) administration of a 0.1-mmol/kg dose of gadoterate meglumine (Dotarem, Guerbet) as well as FLAIR, axial T2-weighted images, diffusion-weighted images, and DSC sequences. ADC maps were generated directly on the scanner. Details on MRI acquisition parameters are available in Supplementary Materials.

### Postprocessing: Anatomical Sequences and ADC

DICOM series were exported and converted to the Nifti format with dcm2niix (https://github.com/rordenlab/dcm2niix). The subsequent processing steps for anatomical sequences were performed using the previously developed processing pipeline of HD-GLIO-AUTO (https://github.com/NeuroAI-HD/HD-GLIO-AUTO);^22,23^ briefly, this included (1) neural-network based brain extraction through the HD-BET^24^ tool (https://github.com/MIC-DKFZ/HD-BET), (2) rigid registration of the image volumes to the native T1-w image using FSL (FMRIB, Oxford, UK), and (3) automated DL based segmentation of the CE and T2-FLAIR components of the tumor using HD-GLIO^22^ (https://github.com/NeuroAI-HD/HD-GLIO). N4 bias field correction and intensity normalization were performed prior to radiomic feature extraction on the anatomical sequences using the ANTsR and WhiteStripe packages (implemented in R v4.1.0, https://github.com/muschellij2/WhiteStripe). ADC maps were generated during the MRI acquisitions by the Syngo software (Siemens Healthcare, Erlangen, Germany) and brain extraction and registration to the corresponding T1-weighted sequence were performed as mentioned above. Median values for ADC maps were calculated both for the CE and T2-FLAIR segmentations with FSL.

### Postprocessing: rCBV Maps

Postprocessing of DSC-weighted MRI data for calculating rCBV maps was performed automatically with the Cercare Medical Neurosuite (Cercare Medical, Aarhus, Denmark). The rCBV maps were then aligned to the corresponding T1-weighted sequence with a rigid registration using FSL. Subsequent processing steps were performed using the *fslstats* and *fslmaths* commands of the same software.^25^ Specifically, to exclude regions of significant signal loss near susceptibility interfaces, such as the skull base or surgical field, a threshold was set on the first volume of the DSC raw data to exclude all values below the 10th percentile of the image intensities distribution. The CE and T2-FLAIR tumor segmentation subsequently used for the evaluation of rCBV parameters were modified accordingly by removing low-signal-intensity regions. Image intensity correction was performed by calculating Gaussian-normalized parameters, as suggested by Ellingson et al.^26^ (the letter “n” was used as a prefix to denote this normalized version of parameter maps, eg, nrCBV). The median values of the nrCBV maps were calculated both for the CE and T2-FLAIR-abnormality masks using segmentations with the previously defined threshold.

### Radiomics Features Extraction

The nonthresholded tumor segmentation masks were used for the subsequent radiomic feature extraction on anatomical sequences—T2-w, FLAIR, T1w-postcontrast—and ADC, and the thresholded segmentation maps were used to calculate radiomics features from nrCBV maps. To reduce dimensionality and considering the multicollinearity of radiomics data, T1w-precontrast sequences were not used to generate radiomics features due to the probable lack of information deriving from this sequence compared to the ones previously mentioned. Radiomics features were calculated using PyRadiomics^27^ (https://pyradiomics.readthedocs.io/) at a binwidth of 25. Shape-based radiomics features (both 2D and 3D) from the original CE and T2-FLAIR tumor segmentation masks were calculated on T2w sequences only. All other intensity-based features, based on the International Biomarker Standardization Initiative^28^ were calculated on all used anatomical sequences, for a total of 938 intensity features for each patient (364 derived from physiologic imaging and 574 derived from anatomical sequences, respectively). The radiomic features (RFs) extracted are listed in the Supplementary Materials.

### Statistical Analysis and Machine-Learning Models

All analyses and ML models were implemented using the R v4.1.0 (R Foundation for statistical Computing, Vienna, Austria). Continuous data are presented as median (interquartile range), and categorical data as number (frequency). Wilcoxon–Mann-Whitney and chi-squared test were used to compare continuous and categorical data, respectively. A *P-*value of <.05 was considered significant for all analyses.

### Radiomics Features Harmonization

Before model development, to counteract the possible batch effect in radiomics features given by the acquisition of MRI studies on different scanners, the Combat feature harmonization technique was implemented through the neuroCombat package^29^ in R (https://github.com/Jfortin1/neuroCombat_Rpackage). The Combat method has already been shown in prior studies to be a valid method to increase the reproducibility of radiomics features.^30,31^ The nonparametric version of neuroCombat was used in order to avoid any assumptions about the distribution of radiomics features.^29^

### Machine-Learning Model Building

Simple generalized linear models based on the median values obtained from the CE and T2-FLAIR segmentation masks of the ADC and nrCBV maps alone, and from a combination of the 2 maps were first constructed.

ML models were then developed using the *caret* package.^32^ To mitigate the problem of class imbalance, as patients with PsPD were less than those with early PD, a synthetic minority oversampling technique was used.^33^

ML algorithms of increasing complexity were trained through the combination of clinical-molecular and/or imaging features by implementing generalized linear models with the least absolute shrinkage and selection operator as an embedded feature-selection method (GLM-LASSO) and using the standard parameters provided by the *caret* package. Briefly, the 4 models were based on:

(i) Clinical data (age, sex, proton therapy [yes/no], surgical procedure (subtotal/gross-total resection), time from the end of RT to the first appearance of an MRI questionable lesion, and MGMT promoter methylation status [methylated/nonmethylated]).
(ii) Clinical data + MGMT promoter methylation status + median values from the ADC and nrCBV maps.
(iii) Clinical data + MGMT promoter methylation status + median ADC/nrCBV data + radiomics features extracted from ADC and nrCBV maps.
(iv) Clinical data + MGMT promoter methylation status + ADC/nrCBV data + radiomics features from both ADC/nrCBV and anatomical sequences (T2, FLAIR, CT1).

Model performances were assessed based on a 5-times repeated 5-fold crossvalidation resampling procedure. The held-out predictions in each of the resampling iterations were used to calculate the areas under the receiver operating characteristic curve, sensitivity and specificity, along with their 95% confidence intervals by using the MLeval package (https://github.com/crj32/MLeval) implemented in R. The ROC curve cutoff point for the calculation of performance measures was automatically chosen as to yield the maximal informedness (informedness = true-positive rate − false-positive rate).^34^ DeLong’s test was used to compare AUC of the models with the highest AUC for each to the simple model built with clinical data alone, which was used as a standard of reference.

### Deep-Learning Models

All analyses were implemented in PyTorch 1.8.1 (Torchvision 0.8.1, CUDA version 11.1). Different variants of the ResNet architecture (ResNet18, ResNet34, ResNet50, ResNet101, WideResNet50, and WideResNet101) were trained using a single 2D image selected at the midpoint between the maximum and minimum z-coordinate of the tumor segmentation volume. ResNet is a DL network architecture that alleviates the problem of creating very deep networks by inserting skip connections between layers and it was shown to outperform previous network architectures in a wide range of image-recognition tasks.^35^ As preprocessing steps, all images were resized to 1 mm^3^ 200 × 200 × 200 voxels and then absolute intensity values were clipped at the 1st and 99th percentile. *Z*-score normalization was then applied, with mean values and standard deviations computed by considering only voxels inside the brain mask. Then, to deal with the small sample of data, the following data augmentation steps were applied: translation of a random value between −20 and +20 pixels along the *x*- and *y*-axis; rotation using a random value between −45 and +45 degrees; cropping to 192 × 192 pixels; contrast variation between 0.7 and 1.5 (all probability per samples = 0.8). After a small, empiric grid search, training parameters were established within the typical standard range. Supplementary Table 1 lists the parameters used for the final training runs.

Model performance was also assessed based on a 5-times repeated 5-fold crossvalidation resampling procedure, and AUC, sensitivity and specificity, along with their 95% confidence intervals were calculated, similarly to the previous ML models.

## Results

After screening a total of 1178 consecutive patients, 105 (8.9%) patients fulfilled the selection criteria and were included in the study, with 85 (80.1%) presenting true progressive disease and 20 (19.9%) presenting PsPD. Table 1 shows the basic demographics, clinical features, and MGMT promoter methylation status of the included patients; no statistically significant differences were recorded between patients with PD and those with PsPD (*P* > .05). OS survival curves for the 2 groups are reported in Supplementary Figure 2.

**Table 1.** Patient Demographic Characteristics

Characteristic	PD, *N* = 85	PsP, *N* = 20	*P*-value^a^
Age, median (IQR)	58 (52, 64)	56 (50, 59)	.2
Sex, *n* (%)			.8
Man	49 (58%)	12 (60%)	
Woman	36 (42%)	8 (40%)	
Surgical procedure, *n* (%)			.3
Complete resection	35 (41%)	11 (55%)	
Partial resection	50 (59%)	9 (45%)	
Proton therapy, *n* (%)	23 (27%)	3 (15%)	.4
Months to PsP suspicion, Median (IQR)	1.22 (0.95, 4.01)	1.13 (0.98, 1.28)	.2
MGMT promoter methylation status, *n* (%)			.2
Methylated	30 (35%)	10 (50%)	
Unmethylated	55 (65%)	10 (50%)	
Corticosteroids at time of MRI			1.0
Yes	65 (76.5%)	15 (75%)	
* *No	20 (23.5%)	5 (25%)	

PD, progressive disease; PsPD, pseudoprogression; IQR, interquartile range; MGMT, O-6-Methylguanine-DNA Methyltransferase.

^a^Wilcoxon rank sum test; Pearson’s chi-squared test; Fisher’s exact test.

**Figure 1.**
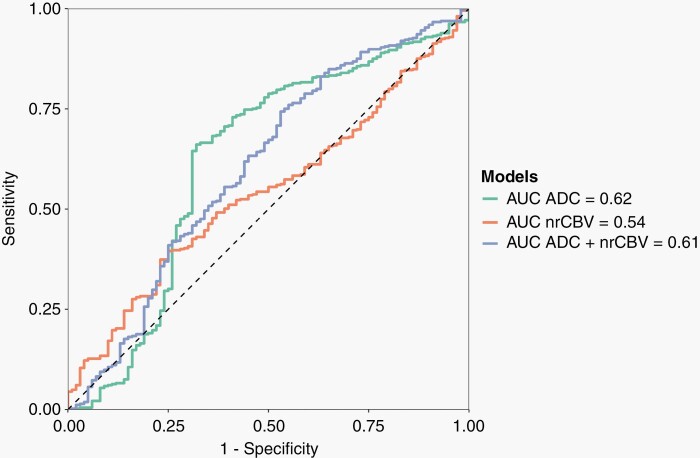
ROC curves for the prediction of pseudoprogression obtained from 3 different models based on simple imaging characteristics (median values of the contrast enhanced and T2-FLAIR segmentations) derived from the apparent diffusion coefficient (ADC, green) and normalized relative cerebral blood volume maps (nrCBV, orange) alone, and for a model combining the median values of both the ADC and nrCBV maps (blue). Overall, all models demonstrated low performance.

The cross-validated AUC for the classification of PsPD based on median ADC values was 0.62 (0.48–0.76), with a sensitivity of 0.75 (0.53–0.89) and specificity of 0.66 (0.55–0.75). A model based on median nrCBV showed a lower AUC of 0.54 (0.31–0.59), with the same sensitivity of 0.66 (0.55–0.75), but lower specificity of 0.35 (0.26–0.46). Combining both maps resulted in an AUC of 0.61 (0.47–0.75), with a sensitivity of 0.65 (0.43–0.82) and specificity of 0.59 (0.48–0.69) (Table 2, Figure 1).

**Table 2.** Diagnostic Performance of Models Based on ADC and nrCBV

Model	SENS (95% CI)	SPEC (95% CI)	AUC-ROC (95% CI)
ADC median	0.75 (0.53–0.89)	0.66 (0.55–0.75)	0.62 (0.48–0.76)
nrCBV median	0.75 (0.53–0.89)	0.35 (0.26–0.46)	0.54 (0.31–0.59)
Combined ADC + nrCBV median	0.65 (0.43–0.82)	0.59 (0.48–0.69)	0.61 (0.47–0.75)

Average performance metrics and 95% confidence intervals of imaging generalized linear models tested with 5-fold repeated crossvalidation. Models based on single parameters from the ADC or nrCBV maps (calculated both for the CE and T2-FLAIR-abnormality masks) and combined ADC + nrCBV do not show reliable discriminatory performances when tested on 5-fold crossvalidation.

ADC, apparent diffusion coefficient; nrCBV, normalized relative cerebral blood volume.

**Figure 2.**
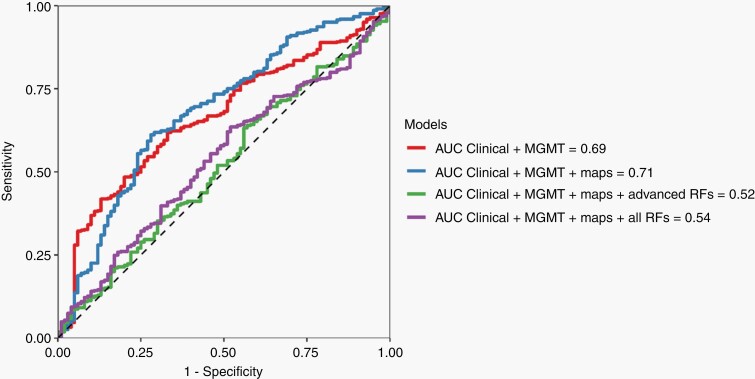
Area under the receiver operating characteristic curve (AUC) of the model including both clinical data and MGMT promoter methylation status and combined clinical-MGMT-radiological models, as listed in the figure legend. Maps refers to the addition to the model of median values from apparent diffusion coefficient (ADC) and normalized relative cerebral blood volume (nrCBV) parametric maps. Advanced radiomics features (RFs) are calculated from ADC and nrCBV sequences. All RFs refers to the addition to the model of radiomics features calculated from all the sequences considered for this study (ADC, nrCBV, T2, FLAIR, and T1-postcontrast). The inclusion of radiomics features determines a significant reduction in model performance compared to a simple model based on clinical-MGMT data alone.

Performance metrics for the generalized linear ML models are summarized in Table 3 and the corresponding ROC curves are depicted in Figure 2. Briefly, a model built on the recorded clinical data and MGMT promoter methylation status yielded an AUC of 0.69 (0.55–0.83), with sensitivity of 0.60 (0.39–0.78) and specificity of 0.75 (0.65–0.83). This model was considered as a reference, and we further refer to it as the reference model. A new model built combining clinical data, MGMT promoter methylation status, median ADC and nrCBV median values slightly increased model performance compared to the reference model, although not significantly (AUC 0.71, 95% CI 0.57–0.85, *P* = .416). Combining clinical data, MGMT promoter methylation status with both median values and radiomics features from ADC and nrCBV maps resulted in a statistically significant lower performance compared to the reference model (AUC of 0.52, 95% CI 0.38–0.66, *P* < .005). The same significant drop in performance compared to the reference model was obtained also when building a model with clinical data, MGMT promoter methylation status, and radiomics features from the anatomical sequences (AUC of 0.54, 95% CI 0.40–0.68, *P* < .005).

**Table 3.** Diagnostic Performance of Increasingly More Complex Generalized Linear Models Based on Clinical and Imaging Data

Model	AUC-ROC (95% CI)	SENS (95% CI)	SPEC (95% CI)	*P*-values^a^
Clinical data + MGMT	0.69 (0.55–0.83)	0.60 (0.39–0.78)	0.75 (0.65–0.83)	Reference
Clinical-MGMT + maps	0.71 (0.57–0.85)	0.85 (0.64–0.95)	0.52 (0.41–0.62)	.416
Clinical-MGMT + maps + advanced RFs	0.52 (0.38–0.66)	0.60 (0.39–0.78)	0.55 (0.45–0.65)	<.005
Clinical-MGMT + maps + all RFs	0.54 (0.40–0.68)	0.55 (0.34–0.74)	0.59 (0.48–0.69)	<.005

The clinical data + MGMT model included the following demographical and clinical variables: age, sex, proton therapy (yes/no), surgical procedure (subtotal/gross-total resection), time from the end of RT to the first appearance of an MRI questionable lesion, and MGMT methylation status (methylated/nonmethylated). Maps refers to the addition to the model of median values from ADC and nrCBV parametric maps. Advanced RFs are radiomics features calculated from ADC and nrCBV sequences. All RFs refers to the addition to the model of radiomics features calculated from all the sequences considered for this study (ADC, nrCBV, T2, FLAIR, and T1-postcontrast).

SENS, sensitivity; SPEC, specificity; AUC-ROC, area under the receiver operating characteristic curve; CI, confidence interval; RFs, radiomic features.

^a^ *P*-values refer to the comparison of AUCs between the current model and the reference clinical-molecular model calculated with DeLong’s test.

In Supplementary Table 3, we report the performance of the same abovementioned models in a subsample of patients, who did not undergo proton therapy. The same inverse trend of reduced model performance when adding radiomics features is seen.

**Figure 3.**
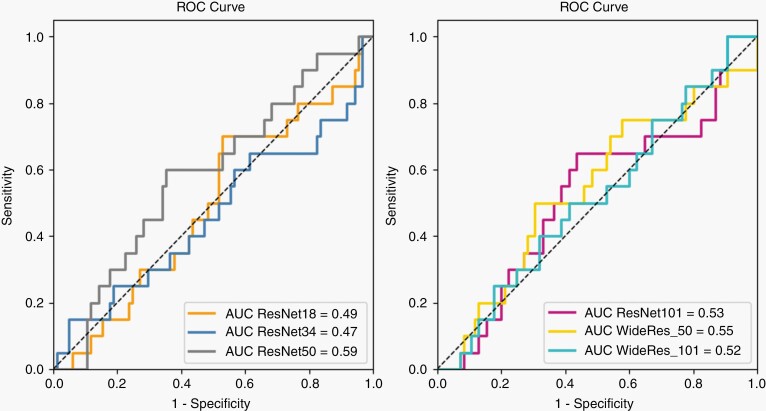
Area under the receiver operating characteristic curve (AUC) of the tested deep learning models based on the ResNet architecture. Models were trained using a single 2D image selected at the midpoint between the maximum and minimum *z*-coordinate of the tumor segmentation volume; data augmentation steps were also included in the pipeline.

DL models were not able to reliably discriminate between PD and PsPD (Figure 3, Supplementary Table 2) and the AUCs for all the tested models tested were ≤ 0.56. The 50 layers-deep convolutional wide neural networks (WideRes-50) achieved the best performance among these types of models, with an AUC of 0.59 (0.49–0.68), sensitivity of 0.88 (0.82–0.94) and specificity of 0.15 (0.08–0.22).

## Discussion

Differentiation of true progression from pseudoprogression still represents a major problem in the follow-up of patients with glioblastoma after chemoradiotherapy. A variety of approaches have been tested to solve this diagnostic dilemma, with no convincing solution up to this day. Here, we tested a stepwise approach by combining methods with increasing complexity. In our study, none of the tested models was able to differentiate pseudoprogression from true tumor progression with sufficient diagnostic performance for satisfactory implementation in clinical practice, despite collecting one of the largest samples published so far on this topic and using a strict patient selection. Simple models based only on ADC or nrCBV values showed low-performance metrics. Furthermore, among all tested ML models, none was found to perform significantly better compared to a simple model based on clinical data and MGMT promoter methylation status alone. Adding to this model also median ADC and nrCBV values from the CE and T2-FLAIR segmentation masks yielded a slightly better predictive performance, but this difference was not statistically significant. Interestingly, when adding radiomics features (both from ADC/nrCBV and from anatomical sequences), the newly obtained models yielded a significantly worse performance compared to models based on clinical data alone.

Our results are in contrast with previously published studies in the literature.^8,14,16,17,21,36^ Several studies have reported promising predictive performances using radiomics on anatomical or physiological imaging techniques (eg, DWI and DSC).^8,14,17,21,36^ However, these investigations often suffered from some methodological limitations which impacted the generalizability of their results, such as small sample size, lack of crossvalidation or absence of independent external testing. In our study, with the use of a larger data sample and crossvalidation on the training set, we achieved an unsatisfactory performance of ADC and nrCBV maps; on unseen data samples, this performance is also likely be lower than what we observed.

ML and DL algorithms represent a promising approach that may help with the diagnostic issue of PsPD, as they allow the generation of high-dimensional models encompassing many layers of data as well as the inclusion of RFs. Here, we created several ML and DL models based on clinical data, MGMT methylation status and imaging using a robust and previously validated method,^29^ which however ultimately lacked an advantage in terms of performance as compared to clinical information alone. Ger et al.^37^ already showed that radiomics features may reduce model performance for head and neck cancer patients, possibly due to the high multicollinearity of data, also when using feature-selection methods. In this regard, a possible explanation is that the addition of radiomics features highly increased the dimensionality of the dataset and introduced random noise that negatively impacted the capability of the model to perform accurate predictions.^38^

Overall, a direct comparison of results between different ML and DL models is often not entirely feasible due to different imaging modalities included in the models, diversified postprocessing methods, and different algorithmic choices; a previous systematic review also demonstrated that reporting standards of radiomics studies in the field of neuro-oncology are currently suboptimal, further limiting reproducibility of results.^39^ Due to this lack of information, it is partially unclear if some of the previous studies on PsPD may have suffered from methodological limitations which ultimately impacted the generalizability of their methods and results. Previous works have reported high AUCs and performance metrics for ML models with different combinations of perfusion and diffusion MRI data,^14,16,17,21,36^ but at the same time they might not be entirely comparable to our results due to lack of crossvalidation or test set, as well as potential data leakage and overfitting due to 2-stage feature selection and high dimensionality of RFs. In our paper, conducted with similar methods but on a different patient population, we found substantially lower performance for our radiomic-based models as compared to previous studies, even when just observing the crossvalidation set, further highlighting the importance of large, multi-centric datasets for the development of more generalizable algorithms.

It is common clinical experience that PsPD is difficult to diagnose based on MRI at the first occurrence of a suspicious contrast-enhancing lesion, even with the aid of DSC or DWI. The accuracy of neuroradiologists to distinguish PD from PsPD at the first occurrence of a questionable lesion on MRI was reported to be as low as 55.8%, with a sensitivity of 69.2% and specificity of 47.1%.^15^ Moreover, the loco-regional brain landscape of patients with glioblastoma treated with chemoradiotherapy may be complex and heterogenous, since PsPD and PD often occur in a disease continuum—with areas of inflammatory reaction mixed with smaller or larger areas of vital/residual tumor—and this may be difficult to diagnose even on histopathological specimens, with mixed responses combining different ratios of inflammation and tumor proliferation found in up to 28% of patients.^8^ Thus, the definition of a robust dichotomous standard of reference for classifying PD versus PsPD may be challenging to achieve both for pathology and for imaging. For these reasons, training supervised artificial intelligence algorithms may represent a challenge, as ground truth data with clear labels may not be easily achievable in practice; at the same time, the low incidence of PsPD and the poor availability of large study samples may further limit the performance of ML algorithms and prevent them to ameliorate the performance of human observers. Our results may thus be a more realistic representation of the current limitations faced by both standard and advanced imaging techniques to overcome the diagnostic dilemma of PsPD and challenge the notion that currently available MRI sequences or models trained on limited data can be fully sufficient for a reliable differentiation of PsPD. However, our methodological pipeline aims at tackling the issue of reproducibility, and almost all the processing steps in our work (apart from the choice of the threshold used for DSC-derived segmentation masks) were performed with automated robust methods that are freely available,^22–24^ with human interference kept to a minimum.

Several limitations apply to our study as well. First, histopathological confirmation of PsPD versus PD was not available for most patients, so the definition of PsPD was based on imaging criteria and follow-up evaluation of patients alone. In this regard, our approach could have led to the incorrect classification of some patients, for example if particularly slow-growing tumor were present in our sample. However, as previously discussed, the presence of an absolute ground truth is debatable even for many histopathological samples. Second, although to the best of our knowledge this is one of the largest sample sizes used to build algorithms to predict PsPD in treated glioblastomas, it is still relatively small for ML or DL models to be effective. We tried to counteract this limitation by performing data augmentation as a preprocessing step before building DL models, but it may still not have been enough to achieve a dataset large enough for valid and robust model training. Furthermore, hyperparameter search was conducted only to a limited amount as a small grid search, and a larger hyperparameter search may also have led to slight improvements in performance. Although these 2 scenarios are certainly possible in principle, vast improvements in model performance with the same amount of training data would not be expected. Moreover, the choice of using the 10th percentile as a threshold to modify masks for the extraction of DSC parameters was based on expert consensus, but other thresholds may have led to better results in our data sample. Another possible limitation is that we decided to not include patients who only underwent biopsy (eg, no surgical treatment) in our sample, given the different volume sizes and the high risk of mixed lesions (eg, only a small proportion of the overall lesion might be PsPD, with the majority still be viable tumor) that could have hindered the classification by the models. Another possible limitation of our study is that we chose to also include patients who underwent proton therapy, as this is a common treatment strategy (25% of patients underwent this type of treatment) in our Center, but we acknowledge that this treatment option might not be available in all Centers and thus limit the generalizability of our results. Nonetheless, we counteracted this possible limitation by building models only on the subsample of patients who did not undergo proton therapy, and we showed that the same trend of reduced model performance when adding radiomics features is observed, and also that model performance is not significantly better compared to that of models trained in the whole cohort. Another possible limitation of our study, common to this research topic, is the possible effect of corticosteroid treatments on image intensity features. Although, it is currently unknown if corticosteroids have a differential effect on radiomics texture features in PD versus PsPD, most patients in our sample (~75%) were not using corticosteroids at the time of first suspicion of PsPD, so it is unlikely that corticosteroid use played any effect in reducing model performance. Lastly, our study did not have a test set. As we ourselves discussed previously, this is mainly required in ML studies in order to evaluate the generalizability of a developed model. However, considering that performance drops from the crossvalidation results are the norm when evaluating unseen data samples, there is no expectation that our models would have performed any better in a separate test set. Therefore, there was no implicit need for an external test in our study after observing the poor performance in the crossvalidation sample. Lastly, our study was focused on the use of MRI, which ultimately presented limited sensitivity and specificity to evaluate disease related processes in the posttreatment setting; other imaging modalities such as molecular or hybrid imaging through positron emission tomography (PET) have been demonstrated to provide more robust imaging biomarkers for the evaluation of gliomas and could potentially improve the diagnostic accuracy in this setting.^40,41^

In conclusion, the diagnosis of pseudoprogression at first occurrence still is a diagnostic challenge for visual evaluation of MR images. We performed a study based on a larger dataset (compared to previous works) using an (almost) fully automatic pipeline and failed to demonstrate a satisfactory performance for models based on ADC or nrCBV or for ML models based on multiparametric MR imaging and radiomics and imaging DL models. Future studies with larger, multicentric cohorts alongside novel or hybrid imaging techniques^42,43^ should be encouraged to develop a better understanding of the real performance and generalizability of supervised ML and DL models in a real life scenario; decentralized techniques for model building such as federated or swarm learning, should also be explored with the aim of building more reliable and clinically translatable models.

## Supplementary Material

vdad016_suppl_Supplementary_Material
