# Supplementary material for: Assessing the added value of apparent diffusion coefficient, cerebral blood volume, and radiomic magnetic resonance features for differentiation of pseudoprogression versus true tumor progression in patients with glioblastoma
Source: Neurooncol Adv. 2023 Feb 21;5(1):vdad016. doi: 10.1093/noajnl/vdad016 (PMC10034916; doi:10.1093/noajnl/vdad016)
Supplement: vdad016_suppl_Supplementary_Material [file vdad016_suppl_supplementary_material.docx]

**Supplementary information**

All data and scripts are available from the Authors upon reasonable request.

**Supplementary Materials and Methods**

*MR acquisition protocol Heidelberg University Hospital*

Sequence parameters for T1 and cT1 MP-RAGE (3D sagittal or axial) were as follows: TI = 900–1100 ms, TE = 3–4 ms, TR = 1710–2250 ms and FA = 15°; for T2 (2D, axial): TE = 85–88 ms; TR = 2740–5950 ms; section thickness, 5 mm; spacing, 5.5mm; for FLAIR (2D, axial): TI = 2400–2500 ms; TE = 85–135 ms; TR = 8500–10 000 ms; section thickness, 5 mm; spacing, 5.5 mm. Diffusion sensitizing gradients were applied sequentially in the x, y, and z directions with b values of 0 and 1200 sec/mm^2^, and corresponding ADC maps were generated on-the-fly by the Syngo software (Siemens Healthcare, Erlangen, Germany). Susceptibility-weighted imaging data were recorded with a three-dimensional fully flow-compensated gradient-echo sequence with the following parameters: 27/19.7, flip angle of 15°, and section thickness of 2.5 mm. Before dynamic imaging a 0.1 mmol/kg prebolus dose of gadoterate meglumine (Dotarem, Guerbet, Roissy, France) was administered in order to reduce T1 effects that might result from contrast agent leakage. DSC imaging was performed with a T2*-weighted gradient-echo echo-planar imaging sequence during the bolus injection of a standard dose (0.1 mmol/kg) of intravenous gadoterate meglumine. Twenty-six to 28 sections with a thickness of 5 mm were acquired with fat suppression (2220/36, flip angle = 90°, field of view = 240 × 240 mm, image matrix = 128 × 128 mm). In total, 50–75 dynamic measurements were performed.

*MR acquisition protocol at DKFZ*

T1- and cT1-weighted sequences (3D sagittal or axial) included both GRE and MPRAGE sequences obtained with the same parameters for each patient. For GRE sequences parameters were as follows: TE = 3.8–4.9 ms, TR = 16– 20 ms and FA = 20-25°, slice thickness = 0.9-1 mm; for MPRAGE sequences, TI = 900 ms. TE = 2.6 ms, TR = 1690–1700 ms and FA = 8°, slice thickness = 1 mm. For T2 (2D, axial) parameters were: TE = 85–109 ms; TR = 4170–6680 ms; slice thickness, 3-4 mm; spacing, 3.3-4.4 mm; for FLAIR (2D, axial): TI =2370–2500 ms; TE = 81–93 ms; TR = 8000-9000 ms; slice thickness, 3 mm; spacing, 3.3-3.9 mm. ADC maps were generated on-the-fly by the Syngo software (Siemens Healthcare, Erlangen, Germany) based on diffusion-weighted imaging acquisitions (b values of 0 and 1000 sec/mm^2^).

After a pre-bolus injection of Gadobenate dimeglumine (Multihance, Bracco, Italy) at a concentration of 0.1 mmol/Kg or gadoterate meglumine (Dotarem, Guerbet, Roissy, France) at a concentration of 0.1 mmol/Kg, dynamic susceptibility contrast images were acquired with 19-25 slices with a slice thickness of 4-5 mm and an inter-slice gap of 5.2-6.5 with fat suppression (TR = 1400-1900 ms, TE = 30-32 ms, flip angle = 90°, image matrix = 128 × 128 mm). In total, 60 dynamic volumes were acquired.

Exclusion criteria (extended):

Exclusion criteria were the presence of the IDH mutation or an unknown IDH or MGMT promoter methylation status (n = 17); the lack of or incomplete information about adjuvant treatment (n = 345); a chemotherapy regimen other than RT + temozolomide/other additional chemotherapy in addition to temozolomide (n = 289); a total RT dose different than 60 Gy (n = 20); imaging performed at external institutions (n = 120); no increase in CE in the first 7 months (n = 95); T2-FLAIR pseudoprogression only without CE increase (n = 18); incomplete availability at the timepoint of first enhancement increase of one or more of the following sequences: pre-contrast T1-weighted, postcontrast T1-weighted, FLAIR, T2-weighted, ADC or DSC MRI sequences (n = 32); heavy motion artifacts (n = 3); corruption of data after conversion to the Nifti format (n = 11); missing or too short follow-up after first enhancement increase (n = 91). Patients who underwent biopsy (n = 32) were excluded due to the intrinsically different macroscopic appearance of the tumor mass.

*List of radiomics features evaluated*

The calculated radiomics features included:

Shape features:

*- MeshVolume - VoxelVolume - SurfaceArea - SurfaceVolumeRatio - Compactness1 - Compactness2*

*- SphericalDisproportion - Sphericity - Maximum3DDiameter - MajorAxisLength - MinorAxisLength*

*- LeastAxisLength - Elongation - Flatness*

First order features:

*- Mean - Variance - Skewness - Kurtosis - Median - Minimum - 10Percentile - 90Percentile - Maximum - InterquartileRange - Range - MeanAbsoluteDeviation - RobustMeanAbsoluteDeviation - Energy - RootMeanSquared - Entropy - Uniformity*

Gray-level co-occurrence matrix features:

*- Autocorrelation - JointAverage - ClusterProminence - ClusterShade - ClusterTendency - Contrast - Correlation - DifferenceAverage - DifferenceEntropy - DifferenceVariance - JointEnergy - JointEntropy - Imc1 - Imc2 - Idm - Idmn - Id - Idn - InverseVariance - MaximumProbability - SumEntropy - SumSquares - SumAverage - SumVariance - Dissimilarity*

Gray level run length matrix:

*- ShortRunEmphasis - LongRunEmphasis - LowGrayLevelRunEmphasis - HighGrayLevelRunEmphasis - ShortRunLowGrayLevelEmphasis - ShortRunHighGrayLevelEmphasis*

*- LongRunLowGrayLevelEmphasis - LongRunHighGrayLevelEmphasis - GrayLevelNonUniformity*

*- GrayLevelNonUniformityNormalized - RunLengthNonUniformity - RunLengthNonUniformityNormalized - RunPercentage - GrayLevelVariance - RunVariance - RunEntropy*

Gray level size zone matrix:

*- SmallAreaEmphasis - LargeAreaEmphasis - LowGrayLevelZoneEmphasis*

*- HighGrayLevelZoneEmphasis - SmallAreaLowGrayLevelEmphasis*

*- SmallAreaHighGrayLevelEmphasis - LargeAreaLowGrayLevelEmphasis*

*- LargeAreaHighGrayLevelEmphasis - GrayLevelNonUniformity*

*- GrayLevelNonUniformityNormalized - SizeZoneNonUniformity*

*- SizeZoneNonUniformityNormalized - ZonePercentage*

*- GrayLevelVariance - ZoneVariance - ZoneEntropy*

Gray level dependence matrix:

*- SmallDependenceEmphasis - LargeDependenceEmphasis - LowGrayLevelEmphasis*

*- HighGrayLevelEmphasis - SmallDependenceLowGrayLevelEmphasis*

*- SmallDependenceHighGrayLevelEmphasis - LargeDependenceLowGrayLevelEmphasis*

*- LargeDependenceHighGrayLevelEmphasis - GrayLevelNonUniformity*

*- GrayLevelNonUniformityNormalized - DependenceNonUniformity*

*- DependenceNonUniformityNormalized - DependencePercentage*

*- GrayLevelVariance - DependenceVariance - DependenceEntropy*

Neighbouring Gray Tone Difference Matrix:

*- Coarseness - Contrast - Busyness - Complexity - Strength*

**Supplementary Figures**


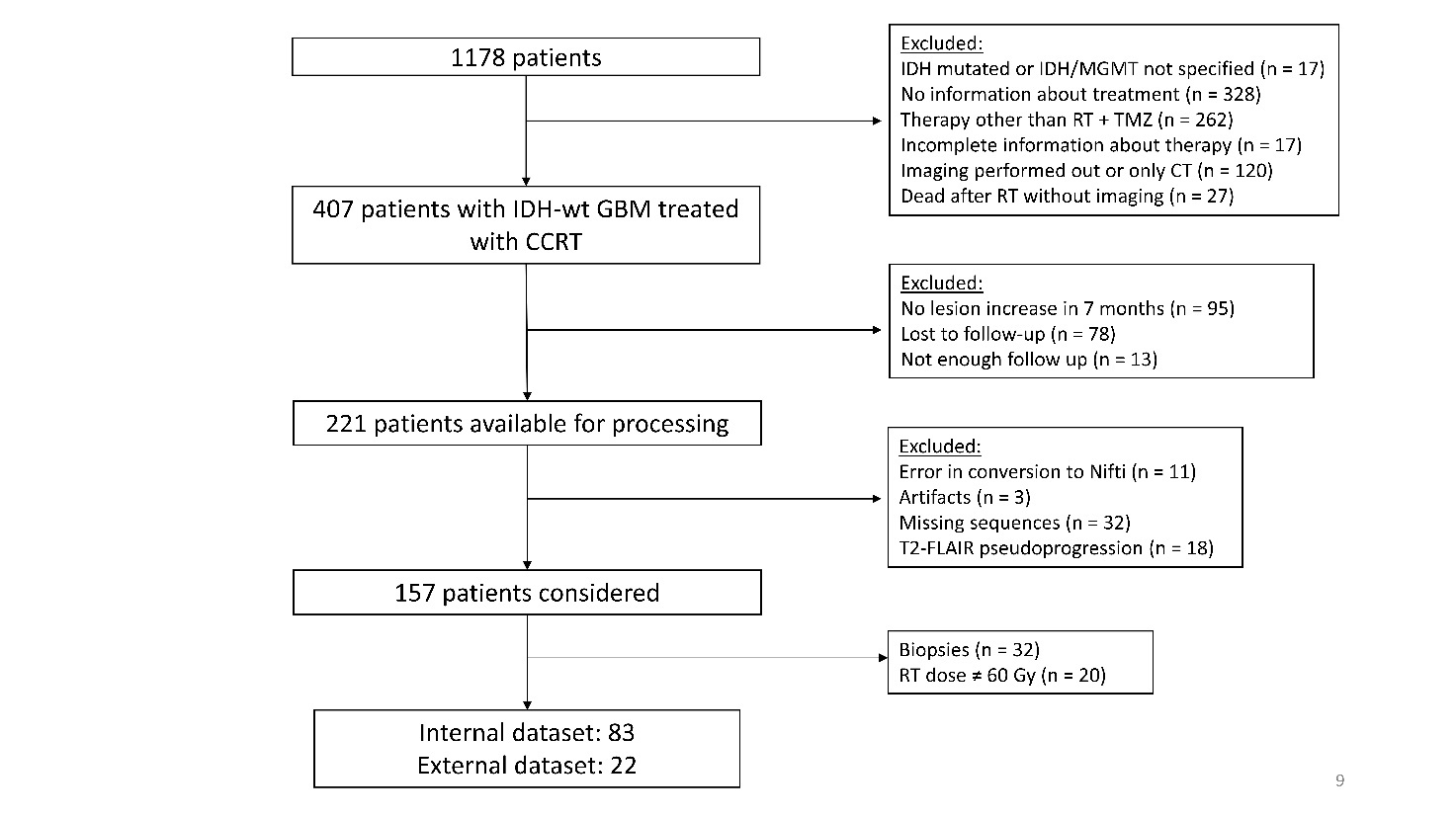
**Supplementary Figure 1.** Patient selection flowchart.

**Supplementary Figure 2.** Kaplan-Meier curves of the Overall Survival of patients with pseudoprogression (PsP) and progressive disease (PD). “p” refers to the p-value of the log-rank test.


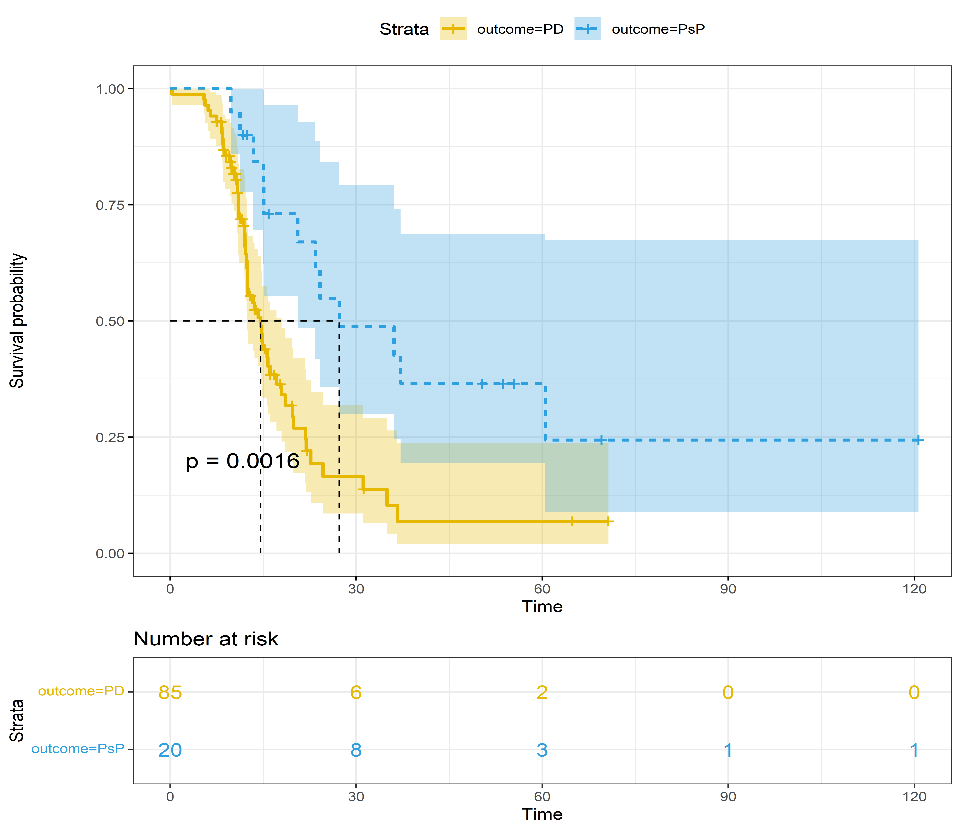


**Supplementary Tables**

| **Model** | **Batch size** | **Optimizer** | **LR** | **Weight decay** | **Momentum** | | **Training length** | **LR scheduler** | | ***gamma*** |
| --- | --- | --- | --- | --- | --- | --- | --- | --- | --- | --- |
| ResNet18 | 32 | SGD | 0.002 | 0.001 | | 0.9 | 250 | 100 | 0.1 | |
| ResNet34 | 32 | SGD | 0.002 | 0.001 | | 0.9 | 250 | 100 | 0.1 | |
| ResNet50 | 32 | SGD | 0.002 | 0.001 | | 0.9 | 280 | 100 | 0.1 | |
| ResNet101 | 32 | SGD | 0.002 | 0.001 | | 0.9 | 280 | 100 | 0.1 | |
| WideRes50 | 32 | SGD | 0.002 | 0.001 | | 0.9 | 280 | 100 | 0.1 | |
| WideRes101 | 32 | SGD | 0.0015 | 0.0001 | | 0.9 | 300 | 120 | 0.1 | |

**S. Table1.** LR scheduler refers to the number of epochs after which the learning rate was reduced to *gamma* * initial learning rate.

SGD = stochastic gradient descent, LR = learning rate

| **Model** | **AUC-ROC (95% CI)** | **SENS (95% CI)** | **SPEC (95% CI)** |
| --- | --- | --- | --- |
| ResNet18 | 0.49 (0.40-0.59) | 0.84 (0.76-0.91) | 0.15 (0.08-0.22) |
| ResNet34 | 0.47 (0.37-0.56) | 0.88 (0.82-0.94) | 0.15 (0.08-0.22) |
| ResNet50 | 0.59 (0.49-0.68) | 0.88 (0.82-0.94) | 0.15 (0.08-0.22) |
| ResNet101 | 0.53 (0.43-0.62) | 0.92 (0.87-0.97) | 0.05 (0.01-0.09) |
| WideRes50 | 0.55 (0.45-0.64) | 0.84 (0.76-0.91) | 0.20 (0.12-0.28) |
| WideRes101 | 0.52 (0.42-0.61) | 0.91 (0.85-0.96) | 0.05 (0.01-0.09) |

**S. Table 2.** Performances of the tested deep learning models built with different variants of the Resnet architecture.

AUC-ROC = area under the receiver operating characteristic curve, SENS = sensitivity, SPEC = specificity, CI = confidence interval.

| Model | AUC-ROC (95%CI) | SENS (95%CI) | SPEC (95%CI) | p-values |
| --- | --- | --- | --- | --- |
| Clinical data + MGMT | 0.63 (0.47-0.79) | 0.94 (0.73-0.99) | 0.48 (0.36-0.61) | Reference |
| Clinical-MGMT + maps | 0.64 (0.48-0.8) | 0.94 (0.73-0.99 | 0.37 (0.26-0.5) | 0.916 |
| Clinical-MGMT + maps + advanced RFs | 0.45 (0.3-0.6) | 0.12 (0.03-0.34) | 0.97 (0.89-0.99) | <0.005 |
| Clinical-MGMT + maps + all RFs | 0.56 (0.4-0.72) | 0.47 (0.26-0.69) | 0.77 (0.66-0.86) | 0.104 |

**S. Table 3. Diagnostic performance of increasingly more complex generalized linear models based on clinical and imaging data for patients who did not undergo proton therapy.**

The clinical data + MGMT model included the following demographical and clinical variables: age, sex, proton therapy (yes/no), surgical procedure (sub-total/gross-total resection), time from the end of RT to the first appearance of an MRI questionable lesion, and MGMT methylation status (methylated/non-methylated). Maps refers to the addition to the model of median values from ADC and nrCBV parametric maps. Advanced RFs are radiomics features calculated from ADC and nrCBV sequences. All RFs refers to the addition to the model of radiomics features calculated from all the sequences considered for this study (ADC, nrCBV, T2, FLAIR and T1-post-contrast).

SENS = sensitivity, SPEC = specificity, AUC-ROC = area under the receiver operating characteristic curve, CI = confidence interval, RFs = radiomic features.

*^a^ P-values* refer to the comparison of AUCs between the current model and the reference clinical-molecular model calculated with DeLong’s test.
